# Do European Union countries adequately address the healthcare needs of adolescents in the area of sexual reproductive health and rights?

**DOI:** 10.1136/archdischild-2019-317073

**Published:** 2019-07-03

**Authors:** Pierre-André Michaud, Annemieke Visser, Johanna Vervoort, Paul Kocken, Sijmen Reijneveld, Mitch Blair, Denise Alexander, Michael Rigby, Martin Weber, Danielle Jansen

**Affiliations:** 1 Lausanne University Hospital, Lausanne, Switzerland; 2 Department of Health Sciences, University of Groningen, University Medical Center Groningen, Groningen, The Netherlands; 3 Department Public Health and Primary Care, TNO, Department Child Health, Leiden University Medical Centre, Leiden, The Netherlands; 4 University Medical Center Groningen, Groningen, The Netherlands; 5 Paediatrics, Imperial College London, Harrow, UK; 6 Imperial College London, London, UK; 7 WHO Regional Office for Europe, WHO, Copenhagen, Denmark

**Keywords:** adolescent, health care, sexual and reproductive health, rights, policy

## Abstract

**Background and objectives:**

Adolescent sexual and reproductive health and rights (SRHR) are of particular relevance given their potential short-term or long-term health consequences. This study evaluates recommendations and policies regarding access to care in this area in 31 European countries (European Union (EU) plus Iceland, Norway and Switzerland).

**Methods:**

As part of the EU funded Models of Child Health Appraised project, data were gathered using a 43-item questionnaire sent to experts responsible for collecting information in each country.

**Results:**

Ten countries have not developed any formal policy or recommendation that guarantee the respect of confidentiality and the possibility of consulting a physician without parents knowing. Nearly half of the countries do not have centres specialised in adolescent healthcare, tackling comprehensive health issues or focusing specifically on SRH. Access to emergency contraception and information regarding pregnancy, including testing, is easy in most countries. However, oral contraception is delivered free of charge in only 10 countries. Twenty-three countries do not meet current standards in terms of providing policy-based pregnancy care, and only 13 have set up special programmes for pregnant adolescents. In only seven countries can adolescents definitely have their pregnancy terminated without their parents knowing (and in another seven countries in selected situations).

**Conclusion:**

The provision and availability of adolescent-friendly SRHR care are far from optimal in around half of the surveyed countries. These results call for the review and implementation of policies, specialised healthcare centres and training initiatives for primary care providers.

**What is already known?**

- Sexual and reproductive health (SRH) constitutes a core part of the health and development of adolescents.
- Sexual behaviour during adolescence can have major health consequences (such as unexpected pregnancy and sexualy transmitted infections (STIs) and psychological sequelae.
- Health professionals, particularly primary care providers and paediatricians, are well placed to respond to adolescents’ needs in the area of SRH.

**What this study adds?**

- In a substantial part of Europe, free access to contraception is limited, as well as healthcare and programmes specifically dedicated to pregnant adolescents.
- Health services providing SRH adolescent-friendly care and respecting adolescents’ rights (eg, confidentiality and autonomy) are not available in nearly half of European Union countries.
- In many situations, health professionals providing SRH to adolescents are not trained to meet adolescents’ SRH needs.

## Introduction

Adolescence, defined as individuals being aged 10–19 years,^1^ is a time to develop one’s identity and acquire autonomy, to explore one’s changing body and sensations and to experiment in numerous areas.^2 3^ Despite its positive aspects, this period of rapid development carries potential negative consequences due to underappreciated risks, especially in the area of sexual and reproductive health.^4^ For instance, in Europe, the rate of pregnancies per 1000 adolescent females, despite recent declines, remains high.^5^ It varies from a low 8‰ in Switzerland to 25‰ in Spain and Portugal and 46‰ in the UK. In most Western European countries, such pregnancies are terminated by abortion (30%–70%), but only around half of these countries provide access to oral contraception (OC) without parental consent, as mentioned in a recently published document of the WHO.^6^ Moreover, 10%–15% of HIV infections are acquired before the age of 20 years.^7^ This has prompted the governments of the European region to endorse an action plan in the area of sexual and reproductive health.^8^

The development of safe and meaningful sexual behaviour depends on many factors that lie beyond the healthcare system, including the existence of comprehensive sexual education or community preventive interventions. However, the quality of care delivered to adolescents plays a pivotal role in the promotion of safe sexual and reproductive health, specifically in the prevention of health-compromising issues such as unplanned pregnancy and STIs.^1 2 9^

It is crucial for healthcare providers to facilitate adolescents’ access to counselling activities and adequate healthcare, to adjust services to their needs and expectations and to respect their rights as they are listed in the UN Convention on the Rights of the Child.^10^ This holds true for health professionals working as primary care providers (including paediatricians in countries in which they play such a role) and for those working in hospitals, for example, emergency and clinical wards. To address these issues, the WHO, along with the UNICEF and the United Nations Population Fund have jointly developed the framework of *adolescent/youth-friendly health services and care*.^11 12^ Interestingly, in many parts of the world, adolescents have been asked about crucial characteristics of such services and care. As a result, both professionals and young people provide a series of recommendations that aim to guarantee the quality of care provided in any setting, such as accessibility, a respectful and supportive attitude by the staff, a comprehensive provision of information and the continuity of healthcare. They stress the importance of interprofessional collaboration and of the respect of adolescents’ rights, such as equity, confidentiality, privacy, as well as their involvement in decisions regarding their health.^13 14^ This paper assesses the extent to which the primary care systems in 31 European countries currently meet existing recommendations in the area of adolescent sexual and reproductive health and rights (SRHR), including the policies, procedures and functioning mentioned above and those proposed by the WHO.^12 15 16^

## Methods

This survey was part of a large European Union (EU)-funded research programme Models of Child Health Appraised) (MOCHA) during 2015–2018, initiated by lead experts from Imperial College in London who cosign this article (MB, DA and MR).^17^ This project, undertaken by several specialist groups of researchers working in collaboration, sets out to assess various aspects of primary care delivered to children and adolescents in all 28 EU countries plus Iceland and Norway, such as quality assessment, economic factors, structure of healthcare delivery, training of healthcare professionals, ethical aspects^18^ and the workforce. The information was collected through several rounds of inquiries and surveys. A network of country respondents (‘experts’) acted as informants for obtaining data requested by the scientists in charge of a given round, using local indigenous sources. These respondents, one for each country, were selected for their expertise in child health services. They were met on several occasions and trained by the MOCHA project’s lead researchers. A detailed description of the methodology of the MOCHA project can be found in the main comprehensive scientific publication from the project^17^; the list of experts can be found on internet (www.childhealthservicemodels.eu/partnerlisting/country_agents/). For the topic of this paper, the first author has added material on the situation in Switzerland to yield a total of 31 countries. All country experts have provided the answer sheet filled in, so that the global response rate of the survey is 100%.

The MOCHA research group on school and adolescent health services developed a 43-item questionnaire focusing on existing policies, recommendations and processes tackling adolescent care, such as the respect of adolescents’ rights, availability of adolescent-friendly services and access to mental and SRHR. In each section, a clinical vignette to exemplify what was expected from the country experts introduced the questions. The first two sections dealt with adolescent-friendly approaches, rights and mental health issues, while the last section tackled specifically SRHR issues. This article focuses mainly on the last section’s content but includes a few topics of other sections that are relevant to SRHR. A list of the questions used for this paper is provided in box 1. All the items in the questionnaire were addressed by the country experts, with very few exceptions (less than 5%). Most answers to the questions were provided as yes/no or using a closed list of specific items (type of professional, location and so on). The answers from each country expert was reported on a large table and then analysed manually, as summarised in tables 1 and 2. The table was sent back to the country experts to make sure that the transcription of the answers was correct. No statistical procedure were used. As this survey does not involve human subjects but rather deals with procedures and policies, it has not been submitted to an institutional review board.

Box 1 **Models of Child Health Appraised round 13 survey’s questions tackling adolescent sexual and reproductive health and rights**

1. In your country, are there specific policies or guidelines for primary care concerning advice on delivering an appropriate, adolescent-friendly service for older children or adolescents? If yes, do the policies or guidelines provide adolescent-specific information about: confidentiality, shared decision making; respect of privacy, provision of information on health and healthy lifestyles and involvement in treatment and adolescent participation?
2. If there are ambulatory facilities for adolescents in your country, do they focus on particular areas of care? Potential answers: sexual reproductive health (contraception, pregnancy tests and counselling), substance misuse, eating disorders and other conditions?
3. Where could an adolescent obtain a pregnancy test? At the local pharmacist, at a retail shop or supermarket, from a primary care physician or a primary care paediatrician, in the emergency ward of a hospital, in a family planning centre (sexual reproductive health counselling centre), in a clinic specialising in adolescent medicine and health, in a ‘community centre’ and other?
4. In your country, if specialised centres for adolescent sexual and reproductive health exist, what do they provide? Information only, counselling and medical care?
5. Can an adolescent consult a family doctor or a gynaecologist without her parents knowing?: yes/no.
6. Is it possible for adolescents to consult a doctor of their choice without their parents’ agreement (ie, chose a doctor other than the one chosen by the parents)?: yes/no.
7. Are condoms easily and readily available to adolescents in your country (available for free or at low cost)?: yes/no.
8. Is oral contraception free of charge to adolescents?: yes/no.
9. Is it possible to get an appointment and a follow-up with a doctor for a drop-out or otherwise vulnerable adolescent such as an uninsured adolescent? Options for answers: the organisation of healthcare gives free access to any adolescent, it will depend on the situation/health problem of the adolescent and no possibility.
10. Is there legislation or policy in your country that guarantees, within any healthcare setting, confidentiality to adolescents (except in life-threatening situations or abuse)? Options: no formal policy/legislation, yes from age 14 or 15 years; yes from age 16–17 years and yes without any age specification (depends on situation/doctor’s opinion).
11. Does the country provide ethical guidelines as how to deal with the assessment of the adolescent’s capacity to make autonomous decisions?: yes/no.
12. In your country, who is able to prescribe or deliver oral and emergency contraception? Options: family doctors, primary care paediatricians, nurse practitioners and school nurses, pharmacists, gynaecologist in primary care and other.
13. Which primary child healthcare professionals would be able to provide advice(s) or counselling for a pregnant adolescent? Options: general practitioner, primary care paediatrician, gynaecologist in primary care, midwife in primary care, nurse/school nurse school doctor, community health worker, social worker and other.
14. Does your country provide specific guidance/standards for primary care professionals with regard to adolescent pregnancy?: yes/no.
15. In your country, are there special programmes or facilities for pregnant adolescents, where they are followed-up until the delivery of the baby? yes/no.
16. Where can an adolescent go to get emergency contraception (the ‘morning after pill’)? Options: community pharmacy; direct access adolescent health or reproductive health clinic, without needing parental consent; hospital emergency department, without needing parental consent; her usual primary care practitioner, without needing parental consent or accompaniment; her normal primary care practitioner but only with parental consent or accompaniment; and other. Emergency contraception is not available.
17. In your country, would it be possible for a 15-year-old adolescent to undergo an abortion without her parents knowing? Options: yes; only if the doctor feels that she is at risk of physical or mental harm; and no.

**Table 1:** Extent to which countries meet available standards in adolescent sexual and reproductive health

	Adol. friendly health service and care	Trained staff (bracket=some)	Available guidelines on confidentiality	Meeting doctor without parents knowing	Free access for vulnerable adolescents somehow	Oral contraception free of charge	Adol. pregnancy: standards for primary care available	Performing a pregnancy test: amount of locations*	Programme for pregnant adolescents	Abortion without parents knowing†
Austria	All topics	No	+	+	+	No	No	2	+	(+)
Belgium	All topics	+	?	+	+	No	No	4	No	(+)
Bulgaria	No	No	No	No	+	No	No	1	+	No
Croatia	All topics	(+)	+	+	+	+	No	3	No	No
Cyprus	No		No	No	No	No	No	1	No	No
Czech Republic	All topics	+	+	+	+	No	No	3	+	No
Denmark	All topics	+	+	+	+	No	+	3	No	(+)
Estonia	Only SRHR	+	+	+	+	No	+	4	+	(+)
Finland	Only SRHR	+	+	+	+	+	+	4	No	+
France	Only SRHR	+	+	+	+	+	+	3	No	+
Germany	No	No	+	+	+	+	No	4	+	+
Greece	All topics	No	+	No	(+)	No	No	3	+	no
Hungary	No	No	No	No	+	No	No	5	No	No
Iceland	No	No	+	+	(+)	No	No	2	No	No
Ireland	No	No	No	+	(+)	No	+	5	+	No
Italy	All topics	+	+	+	+	No	No	4	No	No
Latvia	Only SRHR	(+)	No	+	(+)	No	No	1	+	No
Lithuania	No	No	+	No	(+)	No	No	1	No	No
Luxembourg	All topics	No	+	+	+	No	No	4	No	+
Malta	No	No	+	No	(+)	No	No	2	+	No
The Netherlands	All topics	+	+	+	+	+	No	2	+	(+)
Norway	~No	No	+	+	+	+	No	3	No	(+)
Poland	~No	+	No	No	+	No	+	2	No	No
Portugal	All topics	(+)	+	+	+	+	No	4	+	No
Romania	No	No	No	No	+	+	No	2	No	No
Slovakia	No	No	No	No	No	No	No	1	No	No
Slovenia	All topics	+	No	+	+	+	No	3	No	+
Spain	~No	(+)	+	+	+	No	+	4	+	No
Sweden	Only SRHR	+	+	+	+	+	No	5	No	(+)
Switzerland	All topics	(+)	+	+	+	No	No	5	No	+
UK	All topics	(+)	+	+	+	+	+	5	+	+

*Local pharmacist, supermarket, primary care physician, emergency ward of hospital, family planning centre, adolescent clinic and community centre.

†Brackets: under specific situations only.

**Table 2:** Availability of various professionals to provide emergency contraception (+) and information and care in the area of adolescent pregnancy (*)

	Family doctors	Paediatricians	Nurses	Pharmacists	Gynaecologists	Midviwes	Social workers
Austria	+*	+	*	+	+*	No	*
Belgium	+*	+*	*	No	+*	*	*
Bulgaria	+*		*	No	+*	No	*
Croatia	*	No	No	No	+*	No	No
Cyprus	No	No	No	No	+*	No	No
Czech Republic	No	No	No	No	+*	No	No
Denmark	+*	No	No	+	+*	*	*
Estonia	+	+	No	+	+*	No	No
Finland	+*	*	+*	No	+*	*	*
France	+*	+*	*	+	+*	*	*
Germany	+	+	No	No	+*	*	*
Greece	+*	+	No	No	+*	No	No
Hungary	*	No	No	No	+*	*	*
Iceland	+*	+	No	No	+*	*	*
Ireland	+*	+	No	No	+*	*	*
Italy	+*	+	No	No	+*	*	No
Latvia	+	No	No	No	+*	No	No
Lithuania	+*	No	No	No	+*	*	No
Luxembourg	+*	No	No	+	+*	*	*
Malta	+*	+	No	No	+*	*	*
The Netherlands	+*	No	+	No	+*	*	*
Norway	+*	No	+	No	+*	*	*
Poland	+*	+	No	No	+*	*	*
Portugal	+*	+	+	+	+*	*	*
Romania	+*	+	No	No	+*	*	*
Slovakia	+	+	No	No	+*	No	*
Slovenia	+	+	No	No	+*	No	No
Spain	+*	+	No	+	+*	*	*
Sweden	+	+	+	No	+*	*	*
Switzerland	+*	+	+	+	+*	*	*
UK	+*	+	+	+	+*	+	*

## Results

Altogether, within this specific part of the MOCHA project, a complete set of answers was received either in 2017 or 2018 from all involved countries. The list of the 31 countries is provided in tables 1 and 2.

### Adolescent healthcare services in the field of SRHR (questions 12 4)

As shown in table 1, 18 countries reported the existence of specialised/friendly centres to deliver adolescent healthcare in general. In five countries, these are available in most regions of the country, while in the others, they are present in a limited number of regions. Nearly all of these centres tackle the area of sexual and reproductive health, and many of them (n=13) are broadly oriented (covering SRHR as well as other topics). Most are run by multidisciplinary teams (n=15), but in only 11 countries have the professionals in charge received a formal training in adolescent health (table 1), while in another six, this may be the case in some instances only.

### Adolescents’ rights and ethical issues (questions 6, 9, 10 and 11)

Ten countries have not developed any formal policy or recommendation that guarantee the respect of confidentiality (table 1), and in nine countries, there is no possibility to consult a physician without the parents knowing. Some countries like Norway set a legal age limit for the application of these concepts (often around 15 or 16 years), while in others, like Germany or the UK, it is the responsibility of the physician to decide whether the adolescent is competent enough to be granted professional secrecy. However, except in the UK, no country has developed recommendations pertaining to the assessment of an adolescent’s competence, that is to say, the extent to which he is considered capable of making autonomous decisions regarding his or her health.^19^

In 22 countries, competent adolescents have the possibility to choose a doctor other than the one selected by their parents or caregivers, either for SRH or more generally for other health issues. Some countries provide a specific age limit (usually 15–16 years) for such autonomy, such as Denmark, the Netherlands or Malta. In 23 countries, vulnerable adolescents (eg, street kids, migrants and uninsured youngsters) can get an appointment and follow-up for free, while and in another six countries, it depends on the situation or health problem of the adolescent. Finally, in some countries, such as in Poland or Estonia, when a competent minor adolescent wants to receive healthcare without the parents knowing, he or she has to find a valued and trusted surrogate adult who will accompany her or him in making decisions.

### Providing care in the specific area of SRHR: contraception, pregnancy and abortion (questions 3, 5–8 and 12–17)

Countries vary regarding the provision of SRH services to adolescents. In 20 countries, condoms are easily available to adolescents. They can be provided free of charge in selected facilities (for instance in UK), within the school setting, as free samples after a demo, as part of sexual education sessions (eg, in Denmark), distributed freely to vulnerable populations (eg, in Greece) or at low cost in shops, pharmacies, drugstores and supermarkets. Access to OC is more problematic, as in only a minority of countries (n=11), is it free of charge and often under certain conditions, for example, at a reduced price from 18 to 21 years, like in Norway or the Netherlands. Moreover, in most countries, OC needs to be covered by a prescription. Emergency contraception (EC) can be obtained in several settings across all but three countries. However, as shown on table 2, the number of professionals or institutions that can provide such EC (family doctor, paediatricians, gynaecologists, nurses or pharmacists) differs from one country to another. In countries such as France, Finland, Portugal and Switzerland, adolescents have multiple potential sources, while in others (Croatia, Czech Republic, Hungary and Latvia), EC is available only from family doctors or gynaecologists. One reason for this difference is that in these countries, the adolescent needs a prescription from a doctor, whereas in other countries such as Spain, Sweden and Switzerland, it can be obtained from other professionals without any prescription. In most countries (n=24), no parental consent for EC is required; EC is usually either paid by the insurance or by the adolescent herself or by the parents.

Who can adolescents consult to discuss issues linked with a pregnancy? As expected, a gynaecologist represents an option in all countries (table 2), but in six countries, a family doctor constitutes an alternative. Eight countries have developed recommendations for general practitioners on how to deal with such an issue. In some instances, especially when consulting a doctor implies a bill to the insurance company, adolescents can get advice from nurses (including school nurses), midwives or social workers. In all the countries surveyed, pregnancy tests are easily available (however, not for free), usually in more than one place. Local pharmacies represent the most common resource (n=27), followed by family planning centres (n=17), supermarkets (n=16) and primary care providers (n=13), and in three countries, one can get a pregnancy test at a community centre (Austria, Portugal and Spain). Table 1 gives an idea of the number of different institutions where it is possible to get/perform a pregnancy test. Finally, half of the countries (n=13) have developed special programmes or facilities for pregnant adolescents who want to keep their baby.

In some instances, the situation of a minor adolescent may require the termination of the pregnancy without the parents knowing. As mentioned by the experts from Denmark and Switzerland, this may for instance occur when there is a risk for the adolescent being forced to marry or being sent back to her country of origin or when there is a risk of the girl being molested or seriously beaten by her relatives. In seven countries, such a procedure is feasible, and in another seven countries, it is possible under restrictive conditions. In many instances, according to some experts’ comments, the adolescent should be accompanied by a trusted adult, besides the healthcare professionals in charge (for instance in France, Spain and Switzerland). In several countries such as Bulgaria, Denmark, France, Poland and Sweden, the termination would be covered by the insurance company, while in other countries, for instance in Austria, Estonia, Hungary or Lithuania, the expenses would have to be covered by the adolescent’s parents. In addition, the legal aspects of abortion differ sometimes within a given country, with regulations issued by regional political bodies; in addition, the way the situation is handled also often depends on the opinion of stakeholders such as parents, healthcare providers, or psychologists, whose decisions may be influenced by their own opinions and values.

## Discussion

In summary, only around half of the MOCHA countries have adopted policies or recommendations that aim to promote good access and care in the area of adolescent SRHR; this means that a significant number lag behind the available standards,^12 13 15 16^ including the issue of adolescents’ rights to confidentiality as well as autonomy regarding the choice of a physician. The issue of access to care in the domain of SRH is of particular relevance: while gynaecologists are prepared to deliver good quality care, it is distressing that, in several countries, other healthcare professionals, including paediatricians, do not receive adequate support to address the issue of pregnancy. In 13 countries, health staff meeting young people do not appear to receive adequate training in adolescent medicine. Since the introduction of the concept of ‘gillick competence’,^20^ which deals with the adolescent capacity to make autonomous decisions in the field of SRH, several European countries have issued policies that allow minor adolescents to access contraception (and often abortion as well) (https://fra.europa.eu/en/publication/2017/mapping-minimum-age-requirements/sexual-health-services), without the parents knowing and thus benefit from confidential healthcare. This requires that health professionals have gained expertise in assessing the minor adolescents’ competence and their capacity to make autonomous decisions.^19 20^ In our survey, while many countries support the concept of confidential healthcare, only one provides clear recommendations to professionals as to how to address adolescents’ competence, which is especially problematic in countries that do not provide a legal age limit for granting such competence (for instance in Switzerland). Access to contraception and counselling in the area of SRHR is good in most countries but often not free, and only eight provide recommendations for practitioners to take care of a pregnant adolescent or have developed programmes addressing the situation of pregnant adolescents. The quality of SRH care seems globally problematic in several countries of Central Europe, as shown in table 1.

Some 10 years ago, a survey was run among European countries, which stressed the absence of confidential SRH care in many countries. To a large extent, our results show that little has changed since then. They also reinforce the conclusion of a recently published paper underlining the lack of access to contraception without parental consent in several European countries or reduced access from age 15 or 16 years in others, as well as limited access to abortion—for those under 18 years—without the parents knowing.^6 9^ This is all the more problematic as in 17 EU countries, the minimum age limit for sexual intercourse is set at 14 or 15 years and in another 11 (including Switzerland) countries at age 16 years (https://fra.europa.eu/en/publication/2017/mapping-minimum-age-requirements/sexual-consent). While the European region has endorsed a series of recommendations to meet the standards of the United Nations Convention on the Rights of the Child,^8^ the participation and equal access to SRH care by adolescents is still limited. In addition, while the questions in this survey were focused on females’ SRH, most of the information gathered would also apply to male SRH.

### Strengths and limitations

The interpretation of our results is delicate, as is always the case when it comes to comparing data across countries with different cultures, habits and healthcare systems.^21^ There are thus limitations to this survey. The data presented are based on the report of country experts who have done their best to gather reliable and valid responses but who could be unaware of some existing policies or initiatives taken in their country. For instance, the figures provided by a recent WHO report on the legal access to abortion without parental consent for adolescents under 18 years differs from the information gathered through the MOCHA network.^6^ This divergence may be linked with the fact that the WHO data are delivered by ministries of health, while MOCHA country experts may rather have answered according to the reality faced by health professionals. Such discrepancy stresses the difference between official legal policies on one hand and their implementation in the delivery of healthcare on the other hand. Also, the experts mentioned that the distribution and organisation of the delivery of healthcare differed from one region or one institution to another. Another limitation is that there was no attempt to check the opinion of young people on these issues. This could be a topic of future studies. These limitations do not jeopardise in our view the main conclusion of this survey, which is that much remains to be done until all European countries meet the existing standards of adolescent care in the area of SRHR.

### Implications

There are a number of avenues that could contribute to improving the situation, as exemplified in online supplementary annex 1. Policy makers should strive to implement legal and environmental policies fostering the respect of adolescents’ confidentiality and autonomy that are so crucial in the area of SRH. Policies should also enhance access to healthcare by adolescents facing situations of vulnerability, for instance, in securing the funding of counselling, contraception, pregnancy care and abortion.

10.1136/archdischild-2019-317073.supp1 Supplementary data

Health professionals providing SRH care to adolescents should in the future comply with the standards of adolescent-friendly services and care.^13 22^ This applies to specialised adolescent units where they exist and more broadly to ambulatory settings and hospitals as well as school health services when available. Initiatives in this area should involve adolescents and young people as far as possible, as it is essential to hear their opinion and to be able to meet their specific needs in any cultural and healthcare context.^13^

In addition, given the lack of formal training of professionals working in centres receiving adolescents in the majority of surveyed countries, medical faculties, academic departments and professional organisations should unify their efforts to improve the quality of medical education and care pertaining to adolescent health. This holds true for practitioners working in primary care and for those treating adolescents in hospitals, especially paediatricians. The existence of newly developed standards in the area of medical education as applied to adolescent health should assist them in addressing this challenge.^25 26^
