# Supplementary material for: Do European Union countries adequately address the healthcare needs of adolescents in the area of sexual reproductive health and rights?
Source: Arch Dis Child. 2019 Jul 3;105(1):40–6. doi: 10.1136/archdischild-2019-317073 (PMC6951236; doi:10.1136/archdischild-2019-317073)
Supplement: Supplementary data [file archdischild-2019-317073supp001.pdf]

ANNEX : case studies

Case study on the impact of policies: Switzerland has one of the lowest rate of adolescent pregnancy and abortion. This situation is linked with several factors: a liberal vision of the society towards adolescent sexual and reproductive health, as testified by the 'promotion in schools of condom use and in public areas (posters); policies securing the adolescents' rights and confidential care; comprehensive school-based sexuality education implemented in most public schools; family planning centers widely identified, accessible and used by adolescents.

Case study on the development of adolescent friendly centers: France has developed over the last fifteen years, in most large cities, a series of largely used so-called "maisons des adolescents" (adolescent home) which provide information and support to adolescents – and their families – facing problematic situations such as unplanned pregnancies, victimization or other situations of vulnerability. An interdisciplinary staff including physicians, psychologists and collaborators of social services runs them.

Case study on training in adolescent medicine and health care: Since twenty years, an interdisciplinary group of experts has developed a modular training program called Euteach ("European Training in Effective Adolescent Care and Health" / www.euteach.com). A freely accessible website offers a set of training objectives, slides and teaching tools. Euteach organizes international and regional training courses on adolescent medicine and health that have so far reached around 400-500 participants, many of them being practicing pediatricians.
